# Manipulation of cancer cell pyroptosis for therapeutic approaches: challenges and opportunities

**DOI:** 10.1186/s40364-025-00771-5

**Published:** 2025-04-09

**Authors:** Rui Miao, Xueying Wang, Jingyv Zhang, Qinyv Kang, Qing Liu, Xianglin Luo, Junwei Hou, Baorong Gao

**Affiliations:** 1 Key Laboratory of Birth Defects and Related Diseases of Women and Children of the Ministry of Education, Sichuan University, No. 20, Section 3, Renmin Nan Lu, Chengdu, 610041 China; 2 Department of Obstetrics and Gynaecology, West China Second University Hospital, No. 20, Section 3, Renmin Nan Lu, Chengdu, 610041 China; 3 Department of Otolaryngology Head and Neck Surgery, Central South University, Xiangya Road 87, Changsha, Hunan 410008 China; 4 Otolaryngology Major Disease Research Key Laboratory of Hunan Province, Xiangya Road 87, Changsha, Hunan 410008 China; 5 Xiangya Cancer Center, Xiangya Hospital, Central South University, Changsha, China; 6 Clinical Research Center for Pharyngolaryngeal Diseases and Voice Disorders in Hunan Province, Xiangya Road 87, Changsha, Hunan, 410008 China; 7 National Clinical Research Center for Geriatric Disorders, Xiangya Hospital, Xiangya Road 87, Changsha, Hunan, 410008 China

**Keywords:** Pyroptosis, Gasdermins, Anti-tumor immunity, Cell death, Tumor cells

## Abstract

Remarkable advances have been achieved following discoveries that gasdermins are the executioners of pyroptosis. The pyroptotic process consists a subcellular permeabilization phase and a cell lysis phase, the latter of which is irreversible. Besides immune cells, pyroptosis has also been observed in cancer cells, which exhibit distinct mechanisms compared to canonical immune cell pyroptosis. Although chronic cancer cell pyroptosis fuels tumor growth, intense pyroptotic cell death in tumor cells enhances anticancer immunity by promoting killer lymphocytes infiltration. Triggering pyroptosis in cancer cells is emerging as a promising strategy for cancer treatment. In this review, we introduce the process of cancer cell pyroptosis and its role in antitumor immunity, discuss the translation of these insights into therapies, and highlight current challenges and opportunities in the investigation of cancer cell pyroptosis.

## Introduction

Pyroptosis is a gasdermin-mediated form of regulated necrosis characterized by membrane perforation [1, 2]. The gasdermin family consists of six paralogous genes: GSDMA, GSDMB, GSDMC, GSDMD, GSDME, and Pejvakin (*PJVK*, also known as DFNB59, is a unique member of gasdermin family), features a C-terminal inhibitory domain and an N-terminal cytotoxic domain connected by a flexible linker [1]. Cleavage of the linker by executioner caspases releases the N-terminal domain, which targets and inserts into cellular membranes, initiating non-apoptotic regulated cell death essential for immune responses [3, 4].

The dual roles of cancer cell pyroptosis (CCP) and immune cell pyroptosis (ICP) in tumor immunity create a complex interplay [5]. CCP-induced acute inflammation can activate anti-tumor immunity and inhibit tumor growth, while chronic inflammation from ICP during tumor progression may suppress immunity and promote tumor growth [6, 7]. This highlights the nuanced relationship between pyroptosis and tumor dynamics.

Pyroptosis-induced cancer therapy is an emerging field that leverages pyroptotic cell death for therapeutic purposes. By exploiting features like membrane rupture and inflammatory cascades, this approach selectively targets malignant cells while triggering anti-tumor immune responses. Harnessing pyroptosis’ cytotoxic and immunogenic properties offer potential to overcome tumor resistance and enhance conventional treatments [8–10]. In this review, we explore the principles, applications, and future directions of pyroptosis-induced cancer therapy, highlighting its potential to transform the landscape of cancer treatment modalities.

## Two stages in pyroptotic process: the subcellular permeabilization phase and the cell Lysis phase

**Fig. 1:**
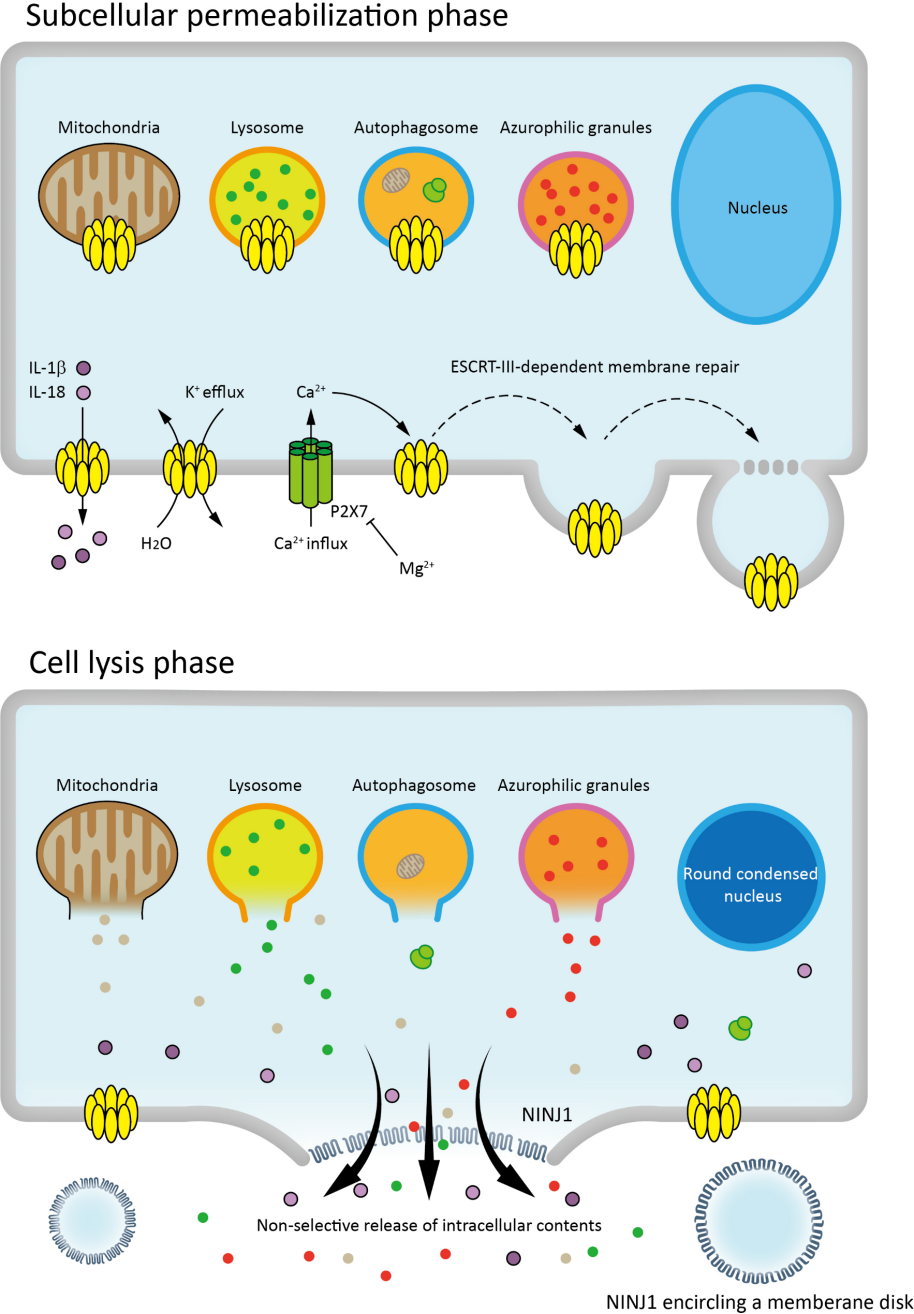
Gasdermin-mediated pyroptosis process. In pyroptosis, the N-terminal domain of gasdermin proteins forms pores in the plasma membrane and organelle membranes (e.g., mitochondria, lysosomes), selectively releasing IL-1β, IL-18, and K^+^. Ca^2+^ influx through P2X7 channels is essential for gasdermin-N oligomerization and pore formation. The subcellular permeabilization phase can be reversed by Mg^2+^-mediated P2X7 inhibition or ESCRT-III-dependent membrane repair, preventing cell death. In the irreversible cell lysis phase, membranes rupture, intracellular contents are non-selectively released, and the nucleus becomes round and condensed

Recent studies have delineated two distinct phases in the pyroptotic cascade: the initial subcellular permeabilization phase and the subsequent cell lysis phase (Fig. 1). Emerging evidence suggests that gasdermin-N domains, liberated upon proteolytic cleavage, are not exclusively inserted into the plasma membrane but are also trafficked to various organelle membranes, including mitochondria, lysosomes, autophagosomes and azurophilic granules [11–14]. Rogers et al. [11] demonstrated that GSDMD- and GSDME-N domains can permeabilize mitochondria membranes, thereby enhancing caspase-3 activation during inflammasome activation. Concurrently, Huang et al. [12] revealed that GSDMD-induced mitochondrial pore formation facilitates the release of mitochondrial DNA into the cytosol, activating the cGAS-STING pathway. While earlier studies implicated caspase-1-mediated GSDMD activation in IL-1β cleavage and release during pyroptosis, recent findings indicate that GSDMD-N domains can permeabilize azurophilic granules and LC3-positive autophagosomes, promoting IL-1β production independently of plasma membrane rupture and pyroptotic cell death [14]. These observations challenge the notion that cell lysis is an inevitable consequence of gasdermin activation during the subcellular permeabilization phase. Instead, gasdermin-mediated organelle permeabilization precedes plasma membrane rupture, and GSDMD-formed plasma membrane pores exhibit greater selectivity than previously assumed [13]. Notably, Evavold et al. [15] demonstrated that GSDMD oligomerizes into plasma membrane pores to facilitate IL-1β secretion from viable macrophages without inducing cell lysis. Recently, endosomal sorting complex required for transport-III (ESCRT-III) machinery has been reported to inhibit pyroptotic cell death downstream of GSDMD activation through membrane repair [16], indicating that pyroptosis in subcellular permeabilization phase is reversible. Similar observation has been reported that magnesium could limit the oligomerization and membrane insertion of GSDMD-N domains by inhibiting the Ca^2+^ channel P2X7 that is required for Ca^2+^ influx and pyroptosis, reversing lipopolysaccharide (LPS)-induced pyroptosis [17]. In the early stage pyroptosis features ion fluxing and IL-1/18 production [13, 15, 18, 19], whereas the cell lysis phase is marked by nuclear condensation, cell swelling due to osmotic imbalance, and ultimately, plasma membrane rupture mediated by Ninjurin-1 (NINJ1) [20–23]. This rupture results in the uncontrolled release of cytosolic and organellar contents [13], culminating in irreversible cell death. Collectively, these findings underscore the complexity of pyroptosis, revealing a dynamic interplay between subcellular events and cell fate.

## Cancer cells have distinct mechanisms of pyroptosis with immune cells

**Fig. 2:**
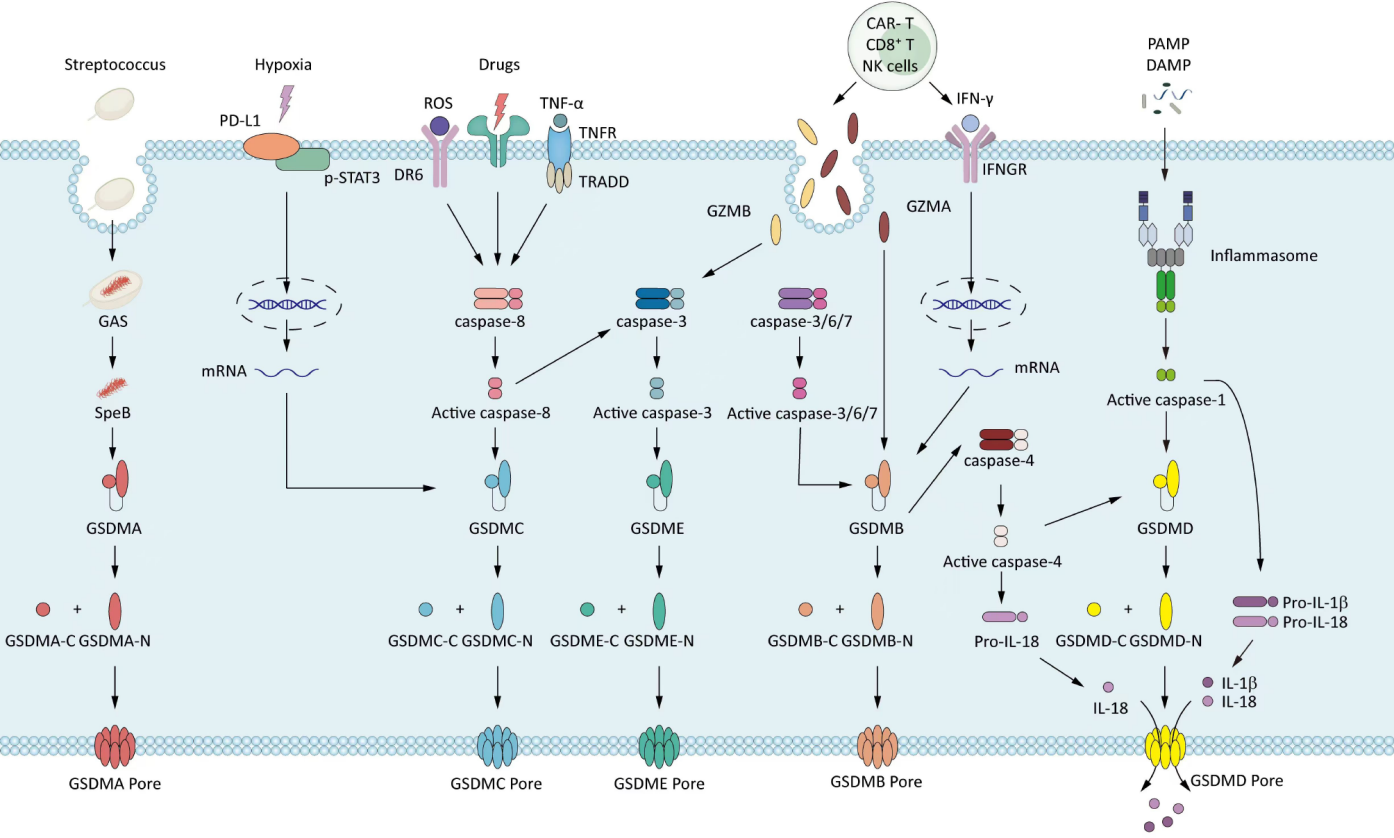
Mechanisms of gasdermin-mediated pyroptosis activation and regulation. SpeB protease cleaves GSDMA at Gln246, forming pores in host cell membranes. Under hypoxia, PD-L1/p-STAT3 (the activated form of STAT3) promotes GSDMC-induced pyroptosis, which is enhanced by TNF-α/CHX but inhibited by STAT3. TNF-α/ROS activate caspase-8 to cleave GSDMC, a process mimicked by certain drugs. GZMB activates caspase-3, cleaving GSDME and converting apoptosis to pyroptosis. GZMA, delivered via perforin, hydrolyzes IFN-γ-upregulated GSDMB, enhancing caspase-4 activity and GSDMD cleavage. Caspase-4 processes pro-IL-18, which is released through gasdermin pores, while H_2_O influx and K^+^ efflux drive pyroptotic cell death

**Fig. 3:**
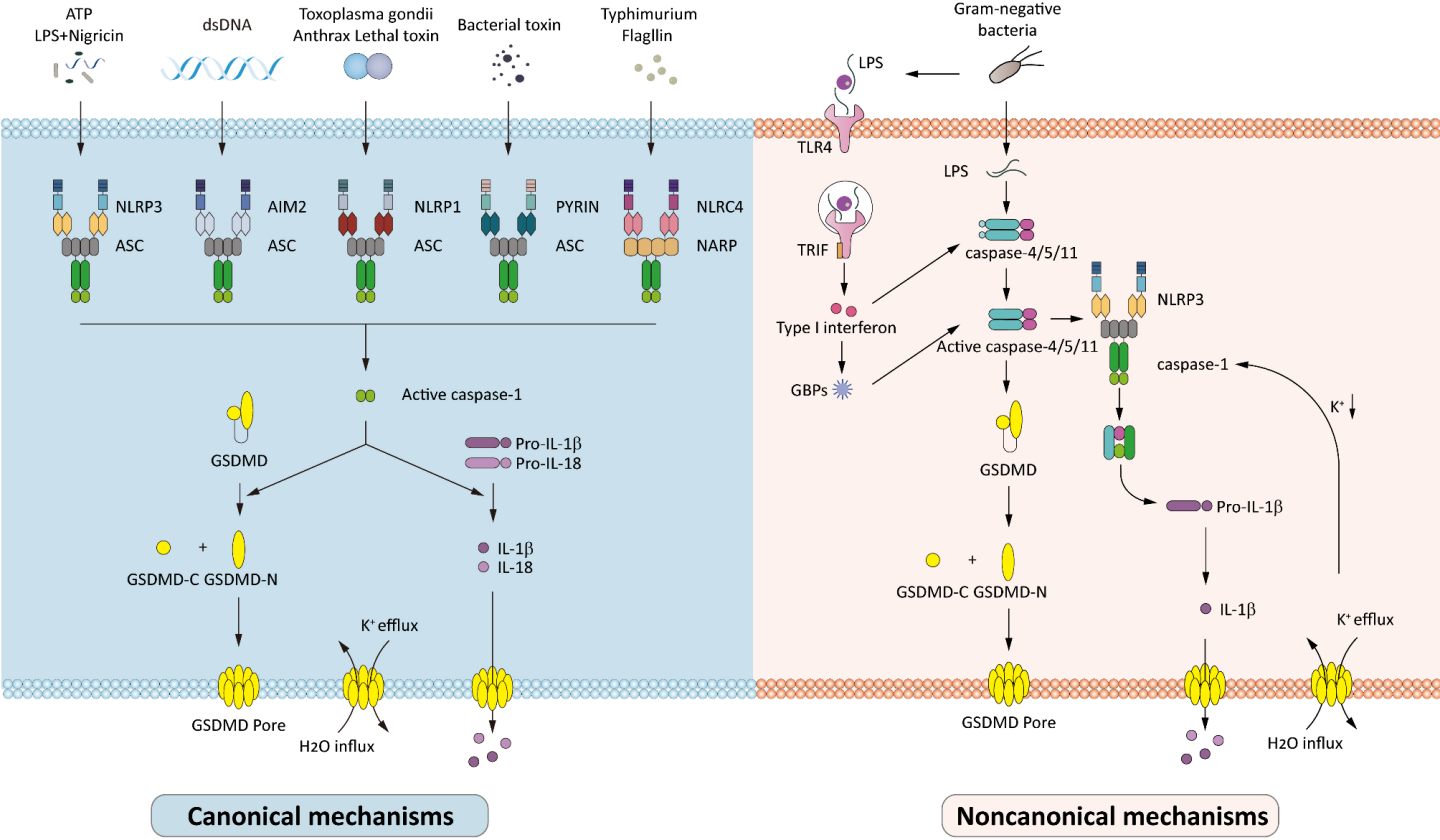
Mechanisms of canonical and non-canonical inflammasome-driven pyroptosis in immune cells. The canonical pathway is a well-orchestrated defense mechanism triggered by infections or cellular damage. When pathogens or danger signals are detected, pattern recognition receptors (PRRs) assemble into inflammasomes (e.g. NLRP3, AIM2, PYRIN, or NLRC4), which activate caspase-1. This enzyme then cleaves pro-inflammatory cytokines, such as IL-1β and IL-18, into their active forms, and also cleaves GSDMD, releasing its pore-forming ability. The non-canonical pathway is a specialized response to intracellular LPS from Gram-negative bacteria, where caspase-4/5/ in human (or caspase-11 in mice) directly binds LPS, bypassing the need for inflammasomes. These caspases cleave GSDMD, enabling it to form membrane pores that cause cell rupture and pyroptosis. Although this pathway does not directly process cytokines, it can indirectly activate the NLRP3 inflammasome

This section highlights the distinct pathways of pyroptosis in immune cells and cancer cells (Fig. 2). Pyroptosis is widely recognized as a host defense mechanism against intracellular pathogens, particularly in macrophages. In canonical pathways, inflammasomes—multimolecular complexes that drive inflammation and pyroptosis—play a central role in orchestrating diverse physiological responses [24, 25]. These inflammasomes, critical components of innate immunity, include the NLRP3 inflammasome (activated by LPS and nigericin), AIM2 inflammasome (triggered by dsDNA such as poly (dA: dT) ), NLRP1 inflammasome (stimulated by Anthrax Lethal toxin or Toxoplasma gondii), PYRIN inflammasome (induced by Clostridium difficile toxin B), and NLRC4 inflammasome (activated by Salmonella enterica serovar Typhimurium or its flagellin) [26–29]. Studies have demonstrated that GSDMD is a key executor of pyroptosis downstream of these inflammasomes [1, 30–32]. Upon activation of pattern recognition receptors (PRRs), inflammasomes catalyze the maturation of caspase-1, a hallmark of the canonical pathway (Fig. 3). In contrast, the noncanonical pathway involves caspase-11, which directly senses LPS from Gram-negative bacteria like Escherichia coli [24, 33–35]. Activated caspase-1/11 can cleave full-length GSDMD into two fragments: N-terminal domain and C-terminal domain [1, 36]. However, only caspase-1 is adept at inducing IL-1β and IL-18 [31, 37, 38].

In cancer cells, pyroptosis is often induced by chemotherapeutic agents that activate caspase-3-mediated cleavage of GSDME [20, 39]. Additionally, apoptosis inducers such as kinase inhibitors, reactive oxygen species (ROS), and cytotoxic granules from killer lymphocytes can trigger pyroptosis in GSDME-expressing cancer cells [40–43]. In hypoxic tumor microenvironments (TME), macrophage-derived TNF-α promotes cancer cell pyroptosis via caspase-8-mediated cleavage of GSDMC [44]. Notably, Zhou et al. [42] revealed that granzyme A (GZMA), released by cytotoxic lymphocytes, directly cleaves GSDMB to induce pyroptosis in cancer cells, underscoring the role of protease-mediated gasdermin activation in this process. Furthermore, Deng et al. [45] and Symmank et al. [46] identified Streptococcal pyrogenic exotoxin B (SpeB) protease as a novel activator of GSDMA, which forms pores in host cell membranes to drive pyroptosis during infection. Collectively, these studies demonstrated that while immune cell pyroptosis is primarily driven by inflammasome activation, cancer cell pyroptosis is often initiated by apoptosis-inducing stimuli, with the proteolytic release of active gasdermin proteins serving as the defining event. This divergence highlights the context-dependent regulation of pyroptosis across cell types and underscores its therapeutic potential in cancer and infectious diseases.

## Introducing cancer cell pyroptosis promotes antitumor immunity

Emerging evidence highlights the dual role of cancer cell pyroptosis in modulating antitumor immunity. Chronic pyroptosis driven by nuclear PD-L1 and GSDMC in hypoxic TME has been shown to promote tumor progression, while acute induction of pyroptosis can stimulate robust immune responses [44]. Wang et al. [9] demonstrated the therapeutic potential of pyroptosis induction using a bioorthogonal system, where nanoparticle-mediated delivery of active gasdermin proteins to 4T1 breast tumor cells enhanced CD3-positive T cell infiltration and synergized with anti-PD-1 therapy to achieve significant tumor regression. Similarly, Zhang et al. [10] reported that granzyme B (GZMB) released by killer lymphocytes cleaves GSDME to induce pyroptosis in cancer cells, promoting phagocytosis by tumor-associated macrophages and increasing intratumoral infiltration of cytotoxic lymphocytes. Further supporting this paradigm, Zhou et al. [42] revealed that GZMA from cytotoxic lymphocytes directly cleaves GSDMB to trigger pyroptosis in GSDMB-expressing cancer cells, thereby amplifying antitumor immunity. Notably, this process is reinforced by a positive feedback loop, as IFN-α and TNF-γ produced by cytotoxic lymphocytes upregulate GSDMB expression in target cells, enhancing tumor clearance in murine models. The immunostimulatory effects of pyroptosis have been partially attributed to the release of damage-associated molecular patterns (DAMPs), such as HMGB1. Studies have shown that BRAF-MEK inhibitor (BRAF-MEKi) promotes GSDME cleavage and HMGB1 release, which activates dendritic cells and increases intratumoral T cell infiltration [8, 47]. Additionally, Tan et al. [48] demonstrated that radiation-induced GSDME-mediated pyroptosis in colorectal cancer cells activates NK cells and enhances antitumor immunity. Clinical observations further support the therapeutic relevance of pyroptosis, as elevated expression of pyroptosis-related molecules correlates with a more immunogenic TME and improved chemotherapeutic responses in bladder cancer patients [49].

Collectively, these findings highlight the potential of harnessing acute pyroptosis to stimulate antitumor immunity, with its targeted induction representing a promising strategy for improving cancer immunotherapy.

## Therapeutically inducing cancer cell pyroptosis for cancer treatment

**Fig. 4:**
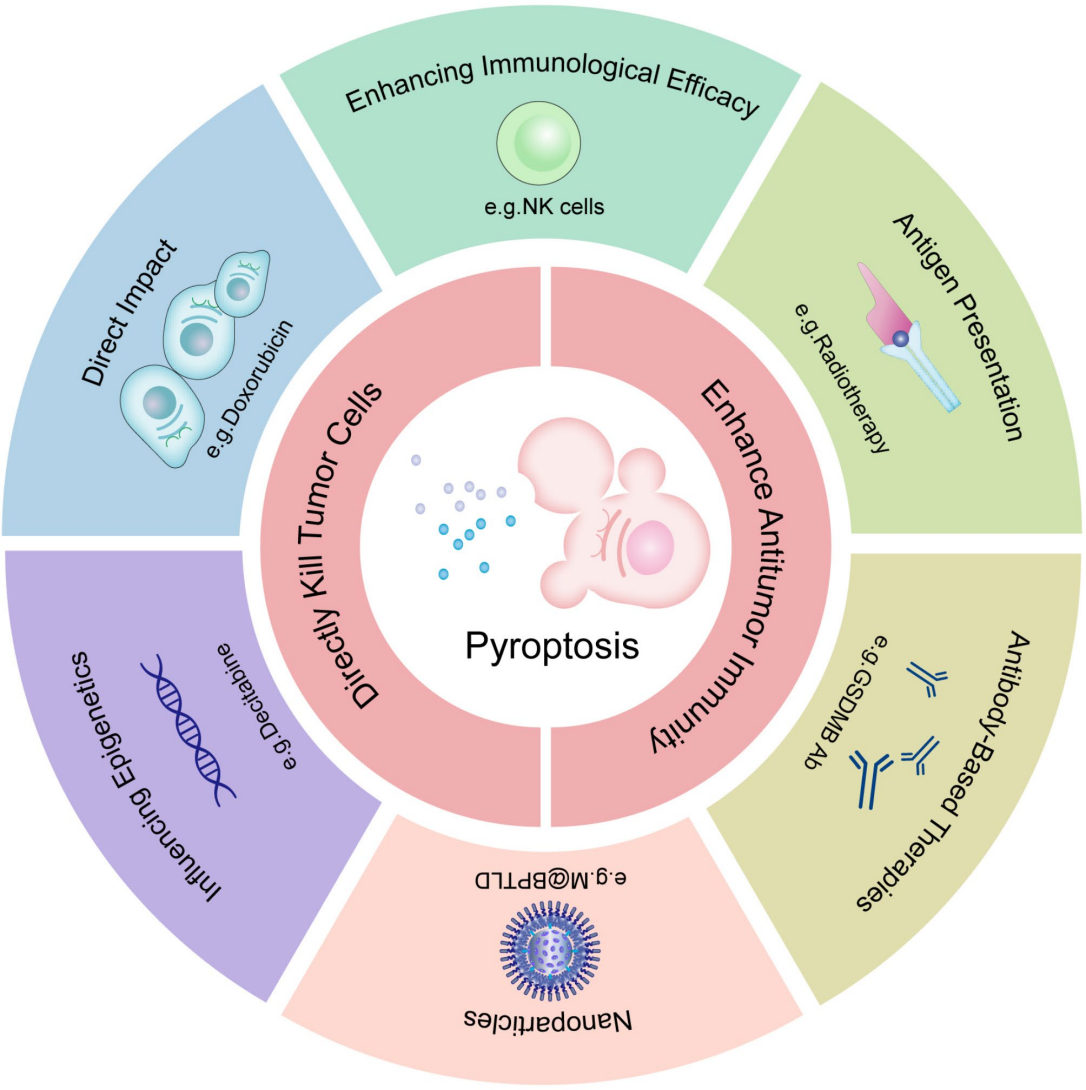
Schematic representation of pyroptosis-related treatment strategies in cancer immunotherapy. Pyroptosis-related treatments are categorized into six groups. These drugs inhibit tumor progression either by enhancing antitumor immunity or by directly killing tumor cells

By leveraging the unique mechanisms of gasdermin activation, researchers have developed targeted approaches to enhance antitumor immunity and overcome resistance to conventional therapies (Table 1; Fig. 4). In 2008, Kim et al. [50] discerned a significant decrease in GSDME mRNA levels in primary breast cancer in comparison to normal tissues, suggesting a tumor-suppressive role. Wang et al. [51] subsequently revealed that various chemotherapeutic agents can induce pyroptosis through the caspase-3/GSDME pathway to treat different tumors. For instance, doxorubicin, cisplatin, and topotecan trigger pyroptosis in various cancer models, including breast cancer (T47D cells), melanoma (MeWo cells), and neuroblastoma (SH-SY5Y cells). Combination therapies further enhance pyroptosis induction. For example, decitabine (DAC) demethylates the GSDME promoter, sensitizing 4T1 breast tumor cells to cisplatin [52]. Similarly, lutetium texaphyrin induces pyroptosis via the caspase-3/GSDME pathway, offering a photochemical approach to cancer treatment [53].

**Table 1:** Summary of pyroptosis-inducing agents and their activation mechanisms in tumor immunotherapy

Agent	Cleavage caspase	Gasdermin profile	Cancer type	Reference
5-FU	caspase-3	GSDME	Gastric cancer	[76]
Actinomycin-D	caspase-3	GSDME	Breast cancer	[51]
Actinomycin-D	caspase-8	GSDMC	Breast cancer	[44]
Actinomycin-D	caspase-3	GSDME	Lung cancer	[51]
AE@ZIF-8 NPs	caspase-3	GSDME	Glioblastoma	[79]
Anthocyanin	caspase-1	GSDMD	OSCC	[77]
As_2_O_3_	caspase-3	GSDME	HCC	[71]
Berberine	caspase-1	N/A	HCC	[73]
BI 2536	caspase-3	GSDME	ESCC	[67]
Bioorthogonal system	N/A	GSDMA3	Breast cancer	[9]
Bleomycin	caspase-3	GSDME	Lung cancer	[51]
Ceritinib	caspase-9	GSDME	Lung cancer	[40]
Cisplatin	caspase-3	GSDME	ESCC	[67]
Cisplatin	caspase-3	GSDME	Breast cancer	[52]
Cisplatin	caspase-3	GSDME	Lung cancer	[59]
Cisplatin	caspase-1	GSDMD	Gastric cancer	[52]
Dasatinib	caspase-3	GSDME	Neuroblastoma/Lung cancer	[39]
Daunorubicin	caspase-8	GSDMC	Breast cancer	[44]
Decitabine	caspase-3	GSDME	Breast cancer	[52]
DHA	caspase-1	GSDMD	Breast cancer	[54]
Diosbulbin-B	caspase-1	GSDMD	Gastric cancer	[52]
Doxorubicin	caspase-3	GSDME	Breast cancer	[51]
Doxorubicin	caspase-8	GSDMC	Breast cancer	[44]
Doxorubicin	caspase-3	GSDME	Lung cancer	[51]
Epirubicin	caspase-8	GSDMC	Breast cancer	[44]
Erlotinib	caspase-3/9	GSDME	Lung cancer	[40]
GNA	caspase-3	GSDME	Colon cancer/Pancreatic cancer/ Breast cancer	[55]
GSDMB Ab	N/A	N/A	Breast cancer	[58]
Inetamab	caspase-1	GSDMB	Lung cancer	[64]
Loratidine	caspase-3/9	GSDME	Lung cancer	[62]
Lutetium texaphyrin	caspase-3	GSDME	Breast cancer	[53]
M@BPTLD	caspase-3	GSDME	Glioblastoma	[80]
Metformin	caspase 1	GSDMD	ESCC/HCC	[69]
Neobavaisoflavone	caspase-3	GSDME	HCC	[72]
Paclitaxel	caspase-3	GSDME	Lung cancer	[59]
Piperlongumine	caspase-1	GSDMD	ESCC	[70]
rMV-Hu191	caspase-3	GSDME	ESCC	[81]
Saikosaponin-D	caspase-1	GSDMD	Lung cancer	[61]
Simvastatin	caspase 1	GSDMD	Colon cancer	[75]
Sophflarine A	caspase-1	GSDMD	Lung cancer	[63]
Topotecan	caspase-3	GSDME	Lung cancer	[82]
Trametinib	caspase-3/9	GSDME	Lung cancer	[40]

Abbreviation: N/A, not available; 5-FU, 5-Fluorouracil; AE@ZIF-8 NPs, biomineralized aloe-emodin with zeolitic imidazolate framework nanoparticles; As_2_O_3_, arsenic oxide; BI 2536, a small molecule inhibitor of mammalian polo-like kinases; DHA, docosahexaenoic acid; ESCC, esophageal squamous cell carcinoma; GNA, gambogeic acid; HCC, hepatocellular carcinoma; M@BPTLD, membrane-encapsulated lonidamine-modified black phosphorus nanosheets; OSCC, oral squamous cell carcinoma; rMV-Hu191, the recombinant Chinese measles virus vaccine strain Hu191

In PD-L1–positive cancer cells, anthracycline antibiotics induce nuclear translocation of PD-L1, upregulating GSDMC expression and activating caspase-8 to cleave GSDMC, thereby triggering pyroptosis [44]. This mechanism is particularly relevant in MDA-MB-231 breast cancer cells, where high levels of GSDMC or PD-L1 correlate with pyroptosis induction. Additionally, gambogenic acid (GNA) derivatives and docosahexaenoic acid (DHA) activate multiple pyroptosis markers in MDA-MB-231 cells, demonstrating broad applicability across cancer types [54, 55].

GSDMB amplification occurs in approximately 60% of HER2-positive breast cancers, where it promotes invasion and metastasis [56, 57]. Molina-Crespo et al. [58] developed a nanomedicine platform utilizing hyaluronic acid–based biocompatible nanocapsules to deliver a specific anti-GSDMB antibody into HER2-positive breast cancer cells. Their findings demonstrated that intracellular delivery of this antibody enhances tumor cell sensitivity, reduces lung metastasis, and inhibits tumor growth in multiple mouse models of HER2-positive breast cancer.

In lung cancer, chemotherapeutic agents such as paclitaxel and cisplatin induce pyroptosis via the caspase-3/GSDME pathway, enhancing the efficacy of PD-L1 inhibitors through the GSDME/IL-12/CD4 + Tem axis [59]. Kinase inhibitors, including ceritinib, trametinib, and erlotinib, also induce pyroptosis by engaging the mitochondrial apoptotic pathway and activating caspase-3/GSDME, however this effect can be shielded by pan-caspase inhibitor Q-VD-OPh [40, 60]. Saikosaponin-D elevates ROS and activates the NF-κB/NLRP3/caspase-1/GSDMD pathway in lung cancer cells [61]. Loratidine enhances PPAR-γ levels, initiating GSDMD transcription and augmenting caspase-8 activation, thereby prompting H1299 or A549 cells to transition from apoptosis to pyroptosis [62]. Sophflarine A, an alkaloid from Sophora flavescens, induces pyroptosis via the ROS-GSDMD pathway, offering a novel therapeutic option for non-small cell lung cancer [63]. Furthermore, a novel anti-HER2 monoclonal antibody inetetamab synergizes with cisplatin, inducing pyroptosis in lung adenocarcinoma and thereby exerting its anticancer efficacy [64].

In melanoma, doxorubicin induces pyroptosis in GSDME-high cell lines, a process enhanced by inactivation of eukaryotic elongation factor 2 kinase (eEF-2 K) [65]. The combination of BRAF and MEK inhibitors is an FDA-approved treatment for BRAFV600E/K-mutant melanoma patients. Smalley et al. [47] proposed that BRAF-MEKi–derived melanoma regression is due to GSDME-induced pyroptosis through HMGB1 release. Melanoma lacking pyroptosis markers is resistant to BRAF-MEKi treatment but sensitive to pyroptosis-inducing chemotherapy, suggesting a proof-of-principle salvage therapy for BRAF-MEKi–resistant melanoma patients. Manipulating the metabolic pathway of cancer cells to initiate pyroptotic cell death is emerging as a promising new strategy for cancer treatment. Elevated iron in melanoma cells initiates ROS signaling, which then drives pyroptosis via a ROS-Tom20-Bax-caspase-GSDME pathway [41]. A low dose of iron supplementation (2 mg/kg) that is used in patients with iron deficiency is sufficient to maximally synergize with the clinical ROS-inducing drugs to curb tumor growth and metastasis of melanoma through GSDME-dependent pyroptosis, suggesting a potential iron-based intervention strategy for melanoma [41, 66].

In esophageal squamous cell carcinoma (ESCC), the PLK1 inhibitor BI2536 synergizes with cisplatin to induce caspase-3/GSDME-mediated pyroptosis [67, 68]. Metformin triggers GSDMD-dependent pyroptosis by targeting the miR-497/PELP1 axis, while piperlongumine inhibits ESCC through the NRF2/ROS/TXNIP/NLRP3 pathway [69, 70]. Several studies have demonstrated that targeted induction of pyroptosis through therapeutic agent delivery into the TME effectively suppresses tumor progression. Hu et al. [71] developed a localized delivery system using arsenic trioxide nanoparticles to treated hepatocellular carcinoma (HCC), which dramatically upregulated the expression of the GSDME N-terminal domain, triggering pyroptosis and resulting in potent tumor growth inhibition in vivo. Neobavaisoflavone influences Tom20 protein expression by generating ROS in HCC cells. This cascade facilitates Bax translocation to mitochondria, subsequent caspase-3 activation, GSDME cleavage, and ultimately pyroptosis in HCC cells [72]. Furthermore, berberine, a natural isoquinoline alkaloid, has been shown to suppress HepG2 liver cancer cell proliferation, invasion, and migration through caspase-1–mediated pyroptosis induction, with consistent effects observed both in vitro and in vivo [73].

In colorectal cancer, lobaplatin was shown to induce pyroptosis in HT-29 and HCT116 cells through a sequential mechanism involving the ROS/JNK/Bax-mitochondrial apoptotic pathway, followed by caspase-3/9 activation and subsequent GSDME cleavage [74]. Similarly, simvastatin triggered pyroptosis in HCT116 and SW620 colorectal cancer cells via the ROS/caspase-1/GSDMD pathway [75]. In gastric cancer research, 5-fluorouracil treatment of SGC-7901 and MKN-45 cells demonstrated the ability to shift cell death mechanisms from caspase-3–dependent apoptosis to pyroptosis [76]. Meanwhile, in oral squamous cell carcinoma, anthocyanin exhibited multi-faceted anti-tumor effects by suppressing cell viability, migration, and invasion through upregulation of NLRP3 and caspase-1 expression, ultimately leading to GSDMD-mediated pyroptosis [77]. In epithelial ovarian cancer cells, α-NETA triggers pyroptosis via the GSDMD/caspase-4 pathway, offering a potential therapeutic strategy for ovarian cancer treatment [78].

Recent studies have explored innovative strategies to induce pyroptosis for cancer therapy. Wang et al. [9] designed a bio-orthogonal system for tumor-specific gasdermin delivery, achieving complete tumor rejection in 4T1 models with only 10-15% of cells undergoing pyroptosis. This approach also sensitizes tumors to checkpoint blockade therapy, highlighting its potential for combination treatments [67]. Fang et al. [79] developed a targeted approach to trigger pyroptosis in glioblastoma, while Ye et al. [80] utilized mitochondria-targeting black phosphorus nanosheets modified with lonidamine to amplify pyroptosis in the same cancer type. Wu et al. [81] employed a recombinant measles virus vaccine to induce pyroptosis in ESCC through caspase-3/GSDME activation, and future studies should focus on optimizing these strategies for clinical translation.

## Challenges and opportunities

Lately, several groups have independently concluded that inducing pyroptosis in cancer cells significantly boosts antitumor immunity. Nevertheless, inconsistencies in experimental results and models need further clarification. For example, Zhang et al. [10] reported that GZMB produced by killer lymphocyte directly cleaves GSDME in a caspase-independent manner to trigger pyroptosis, while Liu et al. [82] found that caspase-3 is essential for GZMB-induced pyroptosis in GSDME-positive target cells, as inhibition of caspase-3 activity prevents this process. In addition, Liu et al. [82] observed that ovalbumin peptide–pulsed GSDME-expressing tumor cells undergo apoptosis, not pyroptosis, when cocultured with murine OT-I T cells. They concluded that high affinity interactions, such as those mediated by chimeric antigen receptor, are necessary for T cells to produce sufficient granzymes to induce pyroptosis.

Zhou et al. [42]. reported pyroptosis in GSDMB-expressing tumor cells pulsed with ovalbumin peptide using the same coculture assay as Liu et al. [82]. They observed GSDMB-positive cells may be more susceptible to killer lymphocyte–triggered pyroptosis than GSDME-positive cells. However, Zhang et al. [10] demonstrated pyroptosis in GSDME-expressing HeLa cells cocultured with NK cells, with pyroptotic cells increasing alongside NK cell numbers. Discrepancies also arise in the classification of cell lines, such as B16 cells, which Zhang et al. [10] considered as GSDME − negative but Liu et al. [82] identified as GSDME-positive. Zhou et al. [10] proposed GZMA cleaves GSDMB to induce pyroptosis [42], while Zhang et al. [10] emphasized GZMB’s role in cleaving GSDME. These conflicting findings underscore the complexity of pyroptosis mechanisms and the need for further clarification.

Translating pyroptosis induction into cancer therapy requires addressing several factors. For instance, pyroptosis in tumor-associated macrophages (TAMs) and myeloid-derived suppressor cells (MDSCs) triggered by pyroptotic tumor cell-derived ATP and DAMPs complicates tumor growth dynamics [31, 83, 84]. IL-1β, a tumor growth promoter, can be secreted by pyroptotic macrophages without cell lysis [15, 85, 86]. Gasdermins can be cleaved by multiple caspases (e.g., caspase-3/6/7/8), and defects in apoptotic pathways may confer resistance to both apoptosis and pyroptosis [44, 51, 87, 88]. Alternative strategies, such as activating non-caspase proteases or delivering active gasdermins directly to tumor cells, could overcome resistance.

Pyroptosis enhances antitumor immunity, but CD8^+^ T cells alone may not suffice; NK cells and macrophages likely play critical roles [8, 82]. HMGB1 and DAMPs released during pyroptosis stimulate dendritic cells and T cells, promoting immunogenic cell death [8, 13]. Combining pyroptosis induction with immunotherapy, such as anti-PD-1 therapy, shows promise, but intrinsic resistance mechanisms must be addressed [9, 16, 17, 42, 89].

A high expression level of gasdermin proteins is crucial for overriding apoptosis [51]. TNF-α and IFN-γ enhance GSDMB expression, sensitizing tumor cells to killer lymphocyte–mediated cytotoxicity, while IRF2 transcriptionally upregulates GSDMD for pyroptosis [38, 82]. Exploring factors that regulate gasdermin expression at transcriptional or protein levels could improve pyroptosis-based tumor elimination. The gasdermin-C domain inhibits the pore-forming activity of gasdermin-N through inter-domain interactions, suggesting that agents disrupting this interaction, such as targeted peptides, could offer an alternative pyroptosis induction strategy [3]. Additionally, crosstalk between pyroptosis and other cell death pathways raises the possibility of inducing pyroptosis by manipulating these pathways, though this remains an open question [90].

For several cancer subtypes, pyroptosis promotes tumor growth and metastasis through inflammatory cytokine release [91–93], activation of oncogenic signaling pathways (e.g., STAT3, PI3K) [94, 95], and dysregulation of gasdermin proteins (e.g., GSDMB, GSDMC) [56, 96]. These mechanisms are tissue-specific and influenced by genetic backgrounds, highlighting pyroptosis as a double-edged sword in cancer biology. Tumor development or clinical treatment and that the clinical outcome depends on the contest between immunosuppressive factors and immune-active factors in pyroptosis.

## Conclusion

Pyroptosis plays a pivotal role in modulating TME and regulating the interactions between tumor and immune cells, making it a critical component in cancer-related activity networks. Recognizing the importance of gasdermins as key executors of pyroptosis in cancer cells, translating experimental findings into clinical applications is essential. However, significant challenges remain. While the introduction of gasdermin-based therapies has shown substantial benefits in treating various cancers, the precise mechanisms by which gasdermins mediate their effects across different cancer types are not fully understood. Additionally, it remains unclear under what conditions gasdermin proteins exert anticancer effects, which biomarkers can predict treatment responses, and which cancer types are most likely to benefit from immunotherapy. Another challenge lies in determining how to effectively induce pyroptosis through combination therapies and whether these therapies should be administered concurrently or sequentially. Furthermore, there is an urgent need to develop pharmacological modulators that can selectively regulate gasdermin activation under specific conditions to optimize therapeutic strategies. Despite these challenges, the current understanding of pyroptosis has significantly advanced cancer therapeutics and paved the way for the development of innovative anticancer treatments.

## Data Availability

No datasets were generated or analysed during the current study.

## Abbreviations

PJVK: Pejvakin
CCP: Cancer cell pyroptosis
ICP: Immune cell pyroptosis
ESCRT-III: Endosomal sorting complex required for transport-III
LPS: Lipopolysaccharide
NINJ1: Ninjurin-1
ROS: Reactive oxygen species
TME: Microenvironments
GZMA: Granzyme A
SpeB: Streptococcal pyrogenic exotoxin B
GZMB: Granzyme B
DAMPs: Damage-associated molecular patterns
BRAF-MEKi: BRAF-MEK inhibitor
DAC: Decitabine
GNA: Gambogenic acid
DHA: Docosahexaenoic acid
eEF-2K: Eukaryotic elongation factor 2 kinase
ESCC: Esophageal squamous cell carcinoma
HCC: Hepatocellular carcinoma
