# Correction: Manipulation of cancer cell pyroptosis for therapeutic approaches: challenges and opportunities

**DOI:** 10.1186/s40364-025-00782-2

**Published:** 2025-04-27

**Authors:** Rui Miao, Xueying Wang, Jingyv Zhang, Qinyv Kang, Qing Liu, Xianglin Luo, Junwei Hou, Baorong Gao

**Affiliations:** 1 Key Laboratory of Birth Defects and Related Diseases of Women and Children of the Ministry of Education, Sichuan University, No. 20, Section 3, Renmin Nan Lu, Chengdu, 610041 China; 2 Department of Obstetrics and Gynaecology, West China Second University Hospital, No. 20, Section 3, Renmin Nan Lu, Chengdu, 610041 China; 3 Department of Otolaryngology Head and Neck Surgery, Central South University, Xiangya Road 87, Changsha, Hunan 410008 China; 4 Otolaryngology Major Disease Research Key Laboratory of Hunan Province, Xiangya Road 87, Changsha, Hunan 410008 China; 5 Xiangya Cancer Center, Xiangya Hospital, Central South University, Changsha, China; 6 Clinical Research Center for Pharyngolaryngeal Diseases and Voice Disorders in Hunan Province, Xiangya Road 87, Changsha, Hunan, 410008 China; 7 National Clinical Research Center for Geriatric Disorders, Xiangya Hospital, Xiangya Road 87, Changsha, Hunan, 410008 China

**Correction: Biomarker Research (2025) 13:58**

10.1186/s40364-025-00771-5

The original article has been amended to display Dr Baorong Gao as the sole Corresponding Author.

